# Investigating the Barriers and Facilitators to Using Antiretroviral Therapy among Women Living with HIV in Plateau State, Nigeria

**DOI:** 10.3390/ijerph21050546

**Published:** 2024-04-26

**Authors:** Emmanuel O. Osayi, Sarah C. Blake, Tolulope Afolaranmi, Oluseye Ajayi, John Onyeji, Atiene S. Sagay, Albert Anderson, Taiwo J. Obindo

**Affiliations:** 1 APIN Public Health Initiatives Jos, Jos 930253, Plateau, Nigeria; 2 Department of Health Policy & Management, Rollins School of Public Health United States of America, Emory University, Atlanta, GA 30322, USA; scblake@emory.edu; 3 Department of Community Medicine, Jos University Teaching Hospital, Jos 930241, Plateau, Nigeria; afolaranmit@unijos.edu.ng; 4 APIN Public Health Initiatives FCT, Abuja 900104, Nigeria; oajayi@apin.org.ng; 5 Faith Alive Foundation Hospital, Jos 930105, Plateau, Nigeria; john.onyeji@binghamuni.edu.ng; 6 Department of Obstetrics and Gynecology, Jos University Teaching Hospital, Jos 930241, Plateau, Nigeria; sagaya@unijos.edu.ng; 7 Department of Medicine, Rollins School of Public Health United States of America, Emory University, Atlanta, GA 30322, USA; aande2@emory.edu; 8 Department of Psychiatry, Jos University Teaching Hospital, Jos 930241, Plateau, Nigeria; obindot@unijos.edu.ng

**Keywords:** women living with HIV, antiretroviral treatment, qualitative research, focus group discussion

## Abstract

Background: Women and girls account for more than 50% of the global HIV population. In Nigeria, the proportion of women living with HIV on long-term antiretroviral therapy (ART) has been on the rise. Despite this, little research exists on their experiences regarding antiretroviral therapy use, especially for women living with HIV (WLHIV) in Plateau State, Nigeria. This study investigates the barriers and facilitators influencing antiretroviral therapy use among women living with HIV. Methods: This study employed a qualitative research design, using focus groups, and included women (female sex workers, pregnant and non-pregnant women living with HIV) and the male partners of serodiscordant couples. Eligibility criteria were being 18 years of age or older, on antiretroviral therapy for more than one year/on pre-exposure prophylaxis (PrEP) for more than one month, and speaking English, Hausa, or both. Data coding utilized both inductive and deductive approaches, and standard content analysis was applied to develop emerging themes. Results: Of the 106 participants, 88 were women living with HIV, and 18 were men in serodiscordant couples. The first facilitator shared by the participants was feeling healthier and stronger due to the antiretroviral therapy, which was also expressed by the male participants on PrEP as feeling good while taking the drug. Additional facilitators shared by the participants included weight gain and having a more positive outlook on life. Participants also disproportionately described barriers to using antiretroviral therapy, including experiences with emotional challenges, physical discomfort, and side effects of ART. Such barriers were linked to feelings of past regret, frustration, and disappointment. Conclusion: This study underscores the significance of maintaining a positive perspective on ART use, demonstrated by the connection between a positive outlook and weight gain, and highlights the hurdles that Plateau State’s women living with HIV face in adhering to antiretroviral therapy. Policymakers and healthcare providers can utilize these findings to formulate targeted strategies aimed at minimizing identified barriers and enhancing antiretroviral therapy utilization among this population via peer- support groups, economic empowerment, and psychosocial support.

## 1. Introduction

Studies have shown that women generally have good health-seeking behavior, so they access HIV testing services and antiretroviral therapy more than men.

According to the United Nations AIDS (UNAIDS) global HIV statistics in 2022, 39 million people were living with HIV, with 53 percent (approximately 21 million) being women and girls 15 years and older [1]. Furthermore, 76 percent (29.8 million) of all the people living with HIV had access to antiretroviral therapy (ART), with 82 percent being women and girls aged 15 years and older [1]. Within twelve years (2010–2022), ART access among people living with HIV significantly increased by 74% (22.1 million) [1,2]. 

In the country, in 2019, the National HIV/AIDS Indicator and Impact Survey (NAIIS) reported an HIV prevalence of 1.4 percent, with a female-to-male ratio of ages 15 and above being 1.9 and 1.1, respectively [3]. Similarly, antiretroviral therapy access among people living with HIV was reported by the National Bureau of Statistics to be higher among women than in men [4,5]. This population of women living with HIV could be further defined by their general characteristics, which are women and HIV (the general population), the highly risky behavior that led to HIV infection (the key population), and their relationship with HIV-seronegative male clients (serodiscordant couples) [6,7,8,9].

The use of ART by people living with HIV (PLHIV), including young girls and women, has significantly transformed the face of the human immunodeficiency virus (HIV) infection in sub-Saharan African countries, including Nigeria, from a terminal illness to a chronic, manageable disease [10,11]. As a chronic, manageable disease, it requires lifelong treatment support for both those who become infected and those affected to cope them with the disease and remain productive. The crucial part of this lifelong treatment support, which requires adherence to antiretroviral therapy, is patient-centered and speaks to the extent to which the behavior of the women living with HIV coincides with their antiretroviral therapy use, which is known to change over time [12].

As the health of women living with HIV improves, the perception of the disease could tilt towards the positive, and they could see the benefit of being on antiretroviral therapy, or it could swing to the negative, leading to life-course events that may decrease adherence to medication [12,13,14]. Hence, there is a need to better understand the barriers to and facilitators of antiretroviral therapy adherence. A meta-analysis and systematic review conducted on 45 studies in sub-Saharan Africa further expressed the need for future research on targeted, specific groups, which include women living with HIV [15]. In Nigeria, a few available studies on the experiences of women living with HIV on their antiretroviral therapy use exist, specifically in Plateau State, Nigeria.

To better understand the perceptions of the community of women living with HIV about the use of lifelong antiretroviral therapy, a focused group discussion was conducted. The focus group discussion is expected to give a good knowledge base of these perceptions and inform the development of an assessment tool to be used for context-specific interventions towards sustaining a continuum of care and antiretroviral therapy treatment among the target population.

## 2. Materials and Methods

### 2.1. Study Setting

This study was conducted in Plateau State, located in the north-central zone of Nigeria. The state is bounded in the north-east by Bauchi State, in the north-west by Kaduna State, in the south-east by Taraba State, and in the south-west by Nasarawa State. It has an area of 26,899 square kilometers and is administratively divided into 17 local government areas (LGAs). The population of the state as of the 2006 census was a total of 3,206,531, with women making up more than 50 percent of the population. The state has a growing population with an annual population change of 2.4 percent and was as be approximately 4,717,300 in 2022 [16].

According to the Nigeria HIV/AIDS Indicator and Impact Survey, the prevalence of HIV among adults aged 15–64 years in Nigeria is 1.4 percent, with prevalence being higher in women than in men [3,17]. Plateau State, in the same survey, had an HIV prevalence of 1.5 percent, slightly higher than the national prevalence, the third highest among states in the north-central zone of Nigeria, which was higher among women. The state was also reported as one of the states in the central and eastern parts of Nigeria, with the local government areas having high antiretroviral therapy coverage [17].

For this study, the Jos North local government area was assumed to be the only urban region in Plateau State, Nigeria, based on the fact that it is the only metropolitan local government in the state with the largest population and has a large amount of developed land [18]. The Jos North local government is reported to have the highest HIV prevalence in Plateau State, significantly higher among women. The Jos East and Jos South local government areas were selected as rural regions in this study for comparative analysis.

### 2.2. Study Design and Participant Selection

This study employed a qualitative research design using focus groups as a major research method. The choice of the study design was to encourage group dynamics among the participants who may have similar but also slightly different experiences with using ART and create rich contextual data on a study topic with little or no prior research.

A purposive sampling method was used in this study. To be part of the focus group, participants were required to be women aged 18 years or older and on antiretroviral therapy for one year or greater. Male partners of serodiscordant couples on pre-exposure prophylaxis (PrEP) for a month or more were also included in this study. All participants were required to speak English, Hausa, or both.

The study participants were first selected by research nurses from the existing records at the HIV clinics where the focus group discussion was conducted. Selected participants were then sorted as HIV-positive female sex workers in the key population [8], pregnant and non-pregnant women living with HIV in the general population [9], and male partners of serodiscordant couples. Based on the study eligibility criteria, 110 participants were enlisted and contacted to explain how and where the focus group discussion would take place. Four (4) women living with HIV (18–24 years of age) and of the pregnant category contacted were removed from the study for not attending the minimum number of focus groups.

### 2.3. Study Procedure

The focus groups were held in Jos North at Hospital 1 and HIV clinic 1, and in Jos East and Jos South, at Hospitals 3 and 4; each focus group lasted an average of one hour. The study groups were led by three moderators, all community health medicine specialists with experience with focus group moderation. The moderators conducted the discussions in Hausa following the semi-structured questionnaire (FGD guide) as shown in Table 1.

It was determined beforehand that a maximum of 12 focus groups could be conducted, after which no new information of value was obtained. At the end of the focus group discussions, each participant was paid 2000 Nigerian naira (NGN), or about USD 2.6, as reimbursement for their transportation expenses and time for participation. All focus group discussions were audio-taped and transcribed verbatim.

### 2.4. Analysis

The audio recordings of the focus group discussions were professionally transcribed. Notes were taken during the focus groups and included in the analysis. We used the qualitative software program Nvivo 12 to manage and code all qualitative data [19]. We used a deductive approach to develop an initial codebook based on the domains of the focus group guide. The codebook was managed in Nvivo 12 and refined to identify inductive codes that emerged from the data. Finally, we applied a constant comparison approach to all deductively and inductively coded data to identify salient themes related to facilitators and barriers using ART therapy for individuals living with HIV in Plateau State, Nigeria.

## 3. Results

Twelve focus group discussions (FGDs) were held between the first and second quarters of 2023: four groups of ten persons, three groups of nine participants, four groups of eight participants, and one group of seven persons (*n* = 106). As shown in Table 2, a total of 106 study participants, including 88 women living with HIV and 18 male partners of serodiscordant couples, participated in the focus group discussions. Most participants (63%) lived in an urban region, while about one-third (37%) of participants lived in a rural region. Most of the participants (64%) were women living with HIV in the general population, about one-fifth (19%) of the participants were female sex workers in the key population, and less than one-fifth (17%) were male partners of serodiscordant couples.

### 3.1. Thematic Findings

We present the key themes from this study as facilitators of and barriers to antiretroviral therapy treatment and pre-exposure prophylaxis. Statements of these findings are presented and supported using illustrative quotes.

### 3.2. Facilitators

There were two major facilitating themes identified by the study participants: feeling healthier and stronger, and positive outcomes and protection.

The first theme, feeling healthier and stronger, was strongly expressed by the women living with HIV and the female sex workers on antiretroviral therapy. As one pregnant participant noted, “The medicine gives me strength” (P8, 37 years), and a female sex worker stated, “the drugs make me healthy, strong and okay” (P4, 35 years). “It will make you healthy and you will not have any problem with your person if you are taking the drugs” (P8, 31 years), “ART drugs make HIV patients look younger, stronger, and more confident, minimizing the negative impact of the virus” (P5, 12 years).

A part of the second theme, protection, was expressed by the male participants on pre-exposure prophylaxis as “*ART will protect you from getting the virus*”.

The women living with HIV in this study added that using antiretroviral therapy protects their serodiscordant couples and keeps them negative, as evidenced by the quote, “*Taking these drugs is good because it helps to protect our husbands from the virus since they don’t have the virus (P7, 21 years)*”. The women living with HIV further stated that the antiretroviral therapy protection extends to their unborn babies during pregnancies, as described in the quote, “*The time I am pregnant for my first daughter, I noticed I have [HIV], but as long as I took my drugs, it protected my baby*” *(P5, 30 years)*. Some of the study participants believed that the positive effects of the use of antiretroviral therapy extend to protection from other illnesses, as shown in the quote, “*it helps to prevent other little illnesses from attacking you*” *(P4, 25 years)*.

In the other part of the second theme, positive outcomes of the use of antiretroviral therapy were expressed in the form of increased appetite that could lead to weight gain among female sex workers, as shown in the quote “*it makes me eat more*” *(P2, 34 years)*.

Additionally, some of the participants noted that antiretroviral therapy has positive effects on the immune system in the quotes “*It helps boost our immune system*” *(P6, 33 years)* and “*The drugs help the immune system, and immediately I take it I feel okay*”. Others believe the positive effects of the use of antiretroviral therapy on the immune system are a result of its effects on the virus, as noted in the quote, “*It helps to fight the virus*” *(P5, 41 years)*. Another study participant noted, “*What I know is that if you are taking the drugs, it renders the virus powerless in the body*” *(P3, 24 years)*. ”*It destroys the strength of the virus*.” *(P4, 25 years)*. “*I understand that it reduces the viral load that we have in the body*”, and “*If you are not taking the drug, your viral load will be normal*”.

The male partners of serodiscordant couples also indicated fewer negative effects of pre-exposure prophylaxis therapy, hence encouraging them to remain on it. One male partner shared, “*so, there is nothing I have felt or the drug made me vomit or anything like that. No but I didn’t feel anything*”.

### 3.3. Barriers

Despite the facilitators and positive effects of using antiretroviral therapy, study participants shared barriers they have encountered. These barriers to using antiretroviral therapy included experiences with negative emotions, physical discomfort, and the side effects of antiretroviral therapy. Negative emotions linked to feelings of frustration were expressed by younger women living with HIV in ab urban region. They disclosed their feelings of discomfort about taking ART medication. One noted: “*Feeling bad because you have to take drugs every day and especially when you are not a drug person*” *(P4, 19 years)*; another said, “*even just seeing the container am feeling awkward already*” *(P9, 17 years); and a third said*, “*Taking the drugs is not easy for me because knowing that I don’t really like drugs, so taking it every day is kind of difficult*” *(P5, 12 years)*.

An older woman living with HIV mentioned that having to use antiretroviral therapy reminds her of her HIV status, which can be upsetting, as shown in the quote, *“because anytime I remember I want to take the drugs, it reminds me of what happened, and it always makes me sad (P6, 40 years)”*.

The older women living with HIV in the urban region also expressed physical discomfort, such as difficulty walking, as stated, “*In fact, at times, I find it very difficult to walk because of my knee; I will go on without eating, thinking I will reduce my weight you know, so that I won’t feel the pain again. That’s the negative aspect*” *(P1, 28 years)*.

The third barrier, antiretroviral therapy side effects such as headaches, dizziness, feeling sleepy, and vomiting, was reported by older women living with HIV, male serodiscordant couples, and female sex workers. Particularly for the female sex workers was the side effect of itching (scratching), as mentioned in the statement, “*not until recently I began to feel some scratches*” *(P6, 25 years)*. The female sex workers also noted side effects of ART like dizziness and vomiting: “*I will feel like vomiting” (P3, 25 years) and “whenever I take the drugs, it makes me feel dizzy, my body will be dizzy*”. Older women living with HIV explained that “*if I take the drug at night I sleep till morning and still continue sleeping into the morning*” *(P6, 40 years)*, while a male participant noted side effects using pre-exposure prophylaxis as “*I feel my head is heavy*” *(P4, 41 years)*.

Some of the antiretroviral therapy side effects could also present as somatic symptoms, such as insomnia and fatigue [20]. These somatic symptoms, reported by studies as related to psychological disturbances and possibly major depression [20], were indicated by older women living with HIV in this study. While those in the urban region reported more difficulty sleeping, a symptom of insomnia, “*since when I started taking the drug when I didn’t know, the drug gives me headache, and I can’t sleep, my eyes I can’t close*”, those in the rural region experienced more fatigue symptoms like heaviness of the head, body weakness, and feeling of dizziness: “*when I started taking it, I was feeling weak*”, “*Me if I take the drugs it makes me feel dizzy*” *and* “*I feel my head is heavy*”.

## 4. Discussion

This study revealed two themes that reflected the positive use of antiretroviral therapy among women living with HIV in Plateau State, Nigeria. These themes included feeling healthier and stronger and having a positive outlook. These themes reflect the participant’s overall well-being (quality of life) using antiretroviral therapy, and their intent to adhere to it could be used to measure the effectiveness of an HIV treatment and care program even at the subpopulation level [21,22,23,24]. The importance of the latter cannot be overemphasized in the study region, where the majority of its women, including those living with HIV (with the extra burden of using ART), housewives, farmers, and the self-employed, are economically struggling to meet their daily demands [25]. This study also showed differences in the intention to adhere to antiretroviral therapy, being greater in the urban than in the rural regions, like the study in Zambia. However, both studies had a higher number of urban participants, which could have contributed to the differences. The enabler of ART adherence, a positive outlook, was notable among FSWs and showed that an all-inclusive key population policy, including being in a group, could influence the intention to adhere to antiretroviral therapy [26]. The latter suggests peer-support groups as an effective strategy for maintaining optimal antiretroviral therapy adherence, in keeping with the findings from a study in Enugu State, Nigeria, by Chime et al. [27].

This study’s findings also identified three negative feelings toward the use of ART among WLHIV in Plateau State, Nigeria: negative emotional reactions, physical discomfort, and side effects of the drug. The negative emotional reactions reported in this study are synonymous with the signs and symptoms of depression, which studies have shown are more common among people living with HIV and even more among women [28,29,30,31]. These depressive symptoms are more likely to be experienced by women, including those living with HIV who are economically disadvantaged [28], which could lead to suicidal ideation and attempts, increased antiretroviral therapy use, or poor adherence to it [30,31,32]. In this study, adolescents and married non-pregnant women reported these depressive symptoms, as expressed in the following quotes: “*Feeling bad because you have to take drugs every day and especially when you are not a drug person*” *(P4, 19 years)*, “*even just seeing the container am feeling awkward already” (P9, 17 years), “Because anytime I remember I want to take the drugs, it reminds me of what happened, and it always makes me sad (P6, 40 years)*”. This is contrary to the findings of the study in OOUTH Sagamu, which found no association between marital status, disclosure of status, and duration since HIV diagnosis [33]. Also, the physical discomfort expressed among older women living with HIV seems consistent with the findings of a systematic review and meta-analysis study by Berner K. et al. 2017 that gait and balance impairment occur in people living with HIV [34]. However, the study added that “the impairments are not influenced by antiretroviral therapy”.

### Strengths and Limitations

A major strength of this study id that the study participants represented different groups of women living with HIV from the general and key populations, which allowed for inclusiveness and obtaining opinions on their perspective on the use of antiretroviral therapy. The defined focus group number for this study was 7–10 participants and was considered adequate [35]. To create a sense of sex equality, male partners of serodiscordant couples were involved in the study. This study has some limitations to consider. For instance, the participants had to remember their antiretroviral therapy use experiences, which might have led to recall bias. Additionally, potential personal biases during the focus group sessions were managed with the help of experienced community health medicine specialists. This study was conducted in Plateau State, Nigeria, and focused on various groups of women living with HIV, making it less generalizable. Qualitative research aims to achieve internal validity and ensures the findings are valid within the studied population. Nonetheless, we are confident the findings can be transferred to other similar studies and populations.

## 5. Conclusions

This study underscores the significance of maintaining a positive perspective on antiretroviral therapy use, as demonstrated by the connection between a positive outlook and weight gain. It also highlights the hurdles that Plateau State’s women living with HIV face in adhering to antiretroviral therapy. Policymakers and healthcare providers can utilize these findings to formulate targeted strategies aimed at minimizing identified barriers and enhancing antiretroviral therapy utilization among this population via peer-support groups, economic empowerment, and psychosocial support.

## Figures and Tables

**Table 1:** Focus group topics and key questions.

Topics	Key Questions
Knowledge of ART	What is the treatment for, and why do you need to take it? How often will you need to take it? What are the side effects of ART you have experienced?
ART medication experiences	What positive feelings do you experience with ART? What negative feelings do you experience with ART? If you have had any negative experiences with ART, what are your coping strategies?
ART Treatment compliance	What makes you forget to take your drug? What are you doing to avoid missing taking your pills?
Access to ART services	What has made you miss your appointment to pick up your ART at the facility? What did you do to prevent you from missing your ART clinic appointment?

**Table 2:** Characteristics of study participants (*n* = 106).

Characteristics	Urban Region Number (*n* = 67)	%	Rural region Number (*n* = 39)	%
**FGD groups**				
WLHIV gen pop	38	56.7%	30	76.9%
WLHIV Key pop	20	29.9%	0	0.0%
partner of a serodiscordant couple	9	13.4%	9	23.1%
**Age disaggregation**				
18–24 years	30	44.8%	10	25.6%
>25 years	37	55.2%	29	74.4%
**Sex**				
Male	9	13.4%	9	23.1%
Female	58	86.6%	30	76.9%
**Duration on ART**				
1–4 years	26	38.8%	4	6.0%
5–9 years	13	19.4%	5	7.5%
≥10 years	19	28.4%	21	31.3%
**Duration on PrEP**				
1–4 months	1	1.5%	4	6.0%
5–11 months	3	4.5%	3	4.5%
≥12 months	5	7.5%	2	3.0%

## Data Availability

All data underlying the findings in the manuscript have be made fully available and hence no repository information to be provided.
